# Garlicnin B1, an Active Cyclic Sulfide from Garlic, Exhibits Potent Anti-Inflammatory and Anti-Tumor Activities

**DOI:** 10.3390/antiox12040869

**Published:** 2023-04-03

**Authors:** Shanghui Gao, Kai Yang, Toshihiro Nohara, Tsuyoshi Ikeda, Jian-Rong Zhou, Kazumi Yokomizo, Jun Fang

**Affiliations:** 1 Faculty of Pharmaceutical Sciences, Sojo University, Kumamoto 860-0082, Japan; gaoshanghui94@gmail.com (S.G.); yangkai@ahyz.edu.cn (K.Y.); none@ph.sojo-u.ac.jp (T.N.); tikeda@ph.sojo-u.ac.jp (T.I.); zhoujr@ph.sojo-u.ac.jp (J.-R.Z.); 2 Department of Medical Technology, Anhui Medical College, No. 632, Furong Road, Hefei 230601, China

**Keywords:** garlicnin B1, anti-inflammation, anti-cancer, natural products, reactive oxygen species

## Abstract

This study aimed to investigate the pharmacological activities of garlicnin B1, a cyclic sulfide compound found abundantly in garlic and structurally similar to onionin A1, which has been shown to possess strong anti-tumor effects. In vitro studies demonstrated that garlicnin B1 significantly reduced intracellular reactive oxygen species triggered by hydrogen peroxide in colon cancer cells. In a mouse colitis model induced by dextran sulfate sodium, garlicnin B1 at a low dose (5 mg/kg) remarkably ameliorated the symptoms and pathological progression. Additionally, garlicnin B1 exhibited considerable tumoricidal activity with an IC50 value of ~20 μM, as observed in cytotoxicity assays. In vivo experiments using the mouse sarcoma S180 transplanted model and the azoxymethane (AOM) or DSS-induced colon cancer model showed that garlicnin B1 effectively suppressed tumor growth in a dose-dependent manner, with marked inhibition at 80 mg/kg. These results suggest that garlicnin B1 has diverse functions that could be achieved by carefully manipulating the dosing regimen. We anticipate that garlicnin B1 has the potential to be used beneficially in the future for the treatment of cancer and inflammatory diseases, although further studies are warranted to elucidate its mechanisms of action.

## 1. Introduction

Despite significant advances in medical technology over the past decades, diseases such as cancer, metabolic disorders, and infectious and inflammatory diseases continue to pose a major threat to human health. As a result, there has been a concerted effort to develop effective preventive and therapeutic strategies. Natural products have gained considerable attention in this regard due to their therapeutic value. For centuries, compounds from plants have been used to treat many diseases and disorders, including cancer [1]. Although many natural products or plant-based medicines have been used as extracts without purification, the isolation and identification of pharmacologically active components are necessary to evaluate their pharmacological effects and to develop natural product-originated therapeutic drugs. In fact, many conventional anti-cancer drugs, such as vinblastine, vincristine, etoposide, and irinotecan, are derived from plants, where the active compounds are first identified in the plants and then synthetic analogs are developed as drugs. Therefore, natural products play a crucial role in the development of therapeutic drugs, offering an opportunity to discover new active compounds as potential drug candidates. Purification and analysis of potential active molecules in plants is a reasonable method for drug discovery with high efficacy and throughput.

Garlic (*Allium sativum*) is an underground bulb belonging to the Liliaceae family. The whole plant, including garlic, garlic leaf, and flower bolt, can be used as a vegetable, seasoning, and medicine. For centuries, garlic has been recognized as a multifunctional plant used for food and medication, especially in traditional Chinese medicine [2]. Garlic contains complex components, including sulfur-containing organic compounds, flavonoids, vitamins, saponins, and polysaccharides. Extensive studies on garlic using fresh garlic extracts, aged garlic, garlic oil, and organosulfur compounds generated by processing garlic have indicated its versatile pharmacological activities, such as strong bactericidal, anti-tumor, anti-aging, blood lipid-reducing, and immune activity-improving effects [3,4]. Thus, isolating and purifying active compounds in garlic have been extensively carried out, and many active garlic components have been identified since the last century. Allicin and alliin were first found in the 1940s, followed by the discovery of volatile components containing a sulfoxide structure [5,6]. Further purification and analysis studies suggested that these allyl sulfur components are the major components in garlic and in other Allium plants that exert pharmacological activity, especially the anti-tumor effect [7,8,9].

In the past decade, Nohara’s group has focused on the isolation, structural characterization, and pharmacological evaluation of sulfides from Allium plants, such as garlic, onion (*Allium cepa*), and Welsh onion (*Allium fisulosum*). They have successfully purified and identified a number of active cyclic sulfides, including onionin A1 and garlicnin B1, where garlicnin B1 is a recently identified, most abundant garlic sulfide [10,11]. Regarding the pharmacological activities of these garlic sulfides, onionin A1 (3,4-dimethyl-5-(1-propenylsulfinyl)thiolane-2-ol) has been extensively investigated in both in vitro and in vivo studies [12,13,14]. A strong tumor-suppressing effect was observed in different solid tumor models, including osteosarcoma and ovarian cancer xenograft models. The activation of antitumor innate immunity is considered to largely contribute to the anti-tumor effect of onionin A1, i.e., inhibiting the activation of tumor-associated macrophages (TAM) [12,13]. These findings suggest the potential of onionin A1 and other active garlic sulfides as adjuvant therapy for cancer patients. Because onionin A1 is the isomer of the major garlic sulfide, garlicnin B1 (3,4-dimethylthiolane S-oxide), it is reasonable to expect that garlicnin B1 may have similar beneficial effects for controlling cancer.

In a recent study, we successfully established an extraction and isolation protocol for garlicnin B1 with a high yield (0.05065%) [11]. We also determined its chemical structure using nuclear magnetic resonance (NMR) spectroscopy with the Mosher method [15,16]. In this study, we investigated the biological activity of garlicnin B1, with a particular focus on its potential in treating cancer. We used two solid tumor models, including a mouse sarcoma S180 transplanted tumor model and a carcinogen-induced mouse colon cancer model (azoxymethane (AOM)/dextran sulfate sodium (DSS)). Additionally, we evaluated the anti-inflammatory potential of garlicnin B1 in a DSS-induced mouse colitis model, considering that many active sulfur-containing compounds exhibit potential anti-oxidative and anti-inflammatory effects [17,18].

## 2. Materials and Methods

### 2.1. Materials

Garlic was purchased from Shinko Company (Kumamoto, Japan). DSS was purchased from MP Biomedicals, LLC (Irvine, CA, USA). AOM, cell culture media (RPMI-1640, DMEM), mouse monocyte chemotactic protein 1 (MCP-1) Quantikine ELISA kit, and mouse tumor necrosis factor-α (TNF-α) Quantikine ELISA kit were purchased from Fujifilm Wako Pure Chemical Corporation (Osaka, Japan). Methyl cellulose, thiazolyl blue tetrazolium bromide (MTT), and 7′-dichlorodihydrofluorescein diacetate (DCDFH-DA) were purchased from Sigma-Aldrich Chemical (St. Louis, MO, USA). Solvents including dimethyl sulfoxide (DMSO), acetone, ethyl acetate (AcOEt), hexane, chloroform, and silica gel 60 were from Fujifilm Wako Pure Chemical Corporation.

### 2.2. Extraction and Purification of Garlicnin B1

The extraction and isolation of garlicnin B1 were conducted using a previously reported method [11] with some modifications. Initially, 2 kg of garlic bulbs were peeled and sliced, and homogenized with 4.2 L acetone, creating a stable environment for allyl sulfenic acid and allyl thio-sulfenic acid, which were derived from the decomposition of allicin. The mixture was left to stand in acetone for 3 days at room temperature. Subsequently, the filtrate was concentrated at 40 °C under a vacuum to obtain a suspension partitioned between ethyl acetate and water. The resulting organic layer was collected and evaporated at 40 °C under vacuum, yielding the raw product containing garlicnin B1. This product was then subjected to column chromatography using silica gel (Φ5 × 30 cm) with the eluent of n-hexane:acetone (6:1 to 1:1, 600 mL of each eluent). The eluate was collected in test tubes (10 mL per tube) and subjected to thin-layer chromatography (TLC), which was performed on silica gel plates (Kieselgel 60 F254, Merck, Rahway, NJ) with standard garlicnin B1 as a reference. The fractions that solely contained garlicnin B1 were collected and evaporated at 40 °C under a vacuum, resulting in the final product of garlicnin B1. The product was further confirmed using TLC on silica gel plates, which were visualized under UV light (254/366 nm) after treatment with 10% H_2_SO_4_ and heating.

### 2.3. UV–Visible Spectrophotometry

UV/visible absorption spectra were recorded on a spectrophotometer (Specord 205 ST, Analytic Jena AG, Germany). Garlicnin B1 was dissolved in acetonitrile/acetone/DMSO (6:3:1) at the concentration of 3 mg/mL, and the absorption spectra were recorded from 200 nm to 800 nm.

### 2.4. Nuclear Magnetic Resonance (NMR)

^1^H NMR spectra were measured using a JEOL ECA 500 NMR spectrometer (JEOL, Tokyo, Japan). The spectra were recorded in the CDCl_3_ solution with chemical shift expressed with tetramethyl silane (TMS) as the internal standard. The chemical shift (δ) was reported in parts per million (ppm).

### 2.5. High-Performance Liquid Chromatography

High-performance liquid chromatography (HPLC) was used to assess the purity of garlicnin B1. A HITACHI Chromaster HPLC system equipped with a Capcell Pak SCX UG80 column (4.6 mmID × 250 mm, Osaka Soda Co., Ltd., Osaka, Japan) and a 5410 UV/visible detector was used. HPLC was performed using an eluent of acetonitrile/acetone/DMSO (6:3:1) with a flow rate of 1.2 mL/min, and the eluate was monitored at 248 nm. The concentration of the sample is 4.5 mg/mL, and 20 μL was applied.

### 2.6. Animals

The male ddY mice (6 weeks old) and male ICR mice (5 weeks old) used in this study were purchased from SLC (Shizuoka, Japan), and they were housed under controlled conditions of 22 ± 1 °C and 55 ± 5% relative humidity with automatic lighting at a 12-h light/dark cycle. All experimental procedures were carried out following the Laboratory Protocol of Animal Handling at Sojo University and were approved by the Animal Ethical Committee at Sojo University (No. 2022-P-023).

### 2.7. In Vivo Anti-Inflammatory Effect of Garlicnin B1

We conducted experiments using a DSS-induced mouse colitis model, in which 2% DSS was administered to the ICR mice via drinking water [19], to assess the anti-inflammatory effects of garlicnin B1. We dissolved garlicnin B1 in methyl cellulose and administered different concentrations of garlicnin B1 (5, 20, 80, and 160 mg/kg) to the mice via intragastric administration three times per week. The control group received methyl cellulose instead of garlicnin B1. During the experiments, we monitored the symptoms and severity of colitis and evaluated them using a semi-quantitative disease activity index (DAI). The DAI was determined by scoring changes in the body weight of the mice, the presence of occult blood, gross bleeding, and stool consistency [20]. We scored weight loss into five grades (0: either a weight gain or no weight loss; 1: 1–5% loss; 2: 5–10% loss; 3: 10–20% loss; and 4: more than 20% loss); stool consistency into three grades (0: normal; 2: loose; and 4: diarrhea); and occult blood into three grades (0: negative; 2: occult blood-positive; and 4: gross bleeding). On the 11th day after administering DSS, when severe symptoms were observed, we sacrificed the mice and collected blood and colon specimens for biochemical and pathological examinations. We used plasma obtained by centrifuging the blood (4000 g, 10 min) to measure inflammatory cytokines, MCP-1 and TNF-α, using an ELISA kit according to the manufacturer’s instructions. Regarding colon specimens, we first measured the length of each colon, then embedded them in O.C.T. compound (Sakura Finetek, Torrance, CA, USA), and froze them at −80 °C. We prepared frozen sections of colon samples (10 μm thick) using a cryostat for histological examination after hematoxylin and eosin (H&E) staining.

### 2.8. In Vivo Anti-Tumor Effect of Garlicnin B1

The anti-tumor effect of garlicnin B1 was investigated in two solid tumor models, the AOM/DSS-induced mouse colon cancer model and the mouse sarcoma S180 subcutaneously transplanted model.

The AOM/DSS model was prepared by injecting AOM (10 mg/kg) intraperitoneally into ICR mice. One week later, the mice were fed 2% DSS in drinking water for one week. By this protocol, multiple tumor nodules in the colon could be found in more than 90% of the mice 10–12 weeks after DSS administration [21,22]. In this model, different doses of garlicnin B1 (5, 10, 20 mg/kg) were administered to the mice three times a week by intragastric administration during the whole experimental period, starting after the injection of AOM. The mice were euthanized 12 weeks after DSS administration, and the colons were collected to measure the number and size of tumor nodules.

The mouse S180 tumor model was prepared by injecting S180 sarcoma cancer cells (2 × 10^6^/100 μL), which were maintained in ddY mouse ascites, into the dorsal skin of ddY mice. At 7 days after tumor inoculation, when the tumor reached 6–8 mm in diameter, garlicnin B1 treatments were carried out at different concentrations (5, 20, 80 mg/kg) three times a week by intragastric administration. During the experiment, tumor regression or growth was observed periodically, and the tumor size was calculated as (W^2^ × L)/2 by measuring the length (L) and width (W) of the tumor. A humane endpoint was established when the size of the tumor reached 4000 mm^3^ or a body weight loss of >10% in the mice.

### 2.9. In Vitro Cytotoxicity of Garlicnin B1

We investigated the cytotoxicity of garlicnin B1 by using mouse C26 colon cancer cells. The cells were cultured in RPMI-1640 containing 10% fetal bovine serum (FBS, Nichirei Biosciences Inc., Tokyo, Japan). The cells were seeded in 96-well plates (3000 cells/well); after overnight preincubation, different concentrations of garlicnin B1 were added to the cells, and the cells were treated for 24 h. The cell viability was then examined by using an MTT assay [23].

### 2.10. Evaluation of the Anti-Oxidative Effect of Garlicnin B1 on C26 Cancer Cells

The C26 cancer cells were seeded in 12-well plates at a density of 2 × 10^5^ cells/well and incubated for 12 h. Different concentrations of garlicnin B1 were then applied to the cells, and the cells were treated for 24 h. The intracellular reactive oxygen species (ROS) probe DCDFH-DA (10 μM) was added to the cells, and they were further cultured for 30 min. During this time, the esterified form of DCDFH-DA permeated cell membranes and was deacetylated to DCDHF, which could react with ROS to produce a fluorescent compound, dichlorofluorescein. Then, the cells were treated with 100 μM hydrogen peroxide (H_2_O_2_) for 30 min. The amount of intracellular ROS was then quantitated by measuring the fluorescence intensity using flow cytometry (BD Accuri^TM^ C6 Plus; Becton Dickinson, San Jose, CA, USA).

### 2.11. Statistical Analyses

All data were presented as means ± standard deviation (SD). Statistical analyses were performed using SPSS version 23.0. Data were analyzed using the analysis of variance (ANOVA) followed by the Bonferroni t-test. A difference was considered statistically significant when the *p*-value was less than 0.05 (*p* < 0.05).

## 3. Results

### 3.1. Purification of Garlicnin B1

**Figure 1:**
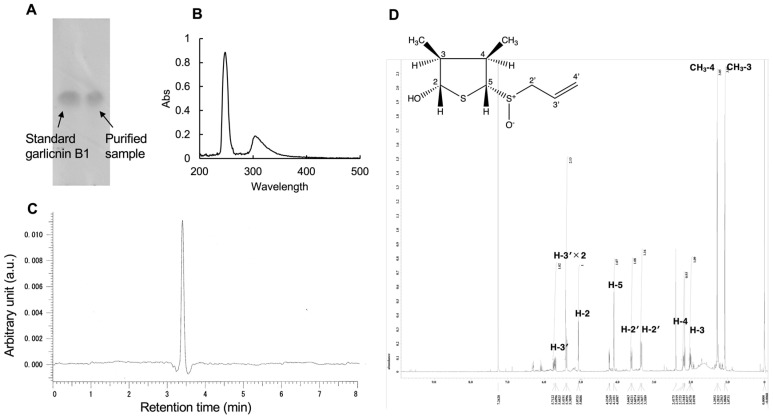
Thin-layer chromatography (TLC) (**A**), UV-vis spectra (**B**), high-performance liquid chromatography (HPLC) (**C**), and ^1^H-NMR spectra (**D**) of garlicnin B1. The TLC analysis showed a single dot, indicating that the garlicnin B1 was relatively pure. In the HPLC analysis, a single peak was observed, indicating the high purity of the compound. ^1^H-NMR spectra were recorded in the CDCl_3_ solution with chemical shift expressed with tetramethyl silane (TMS) as the internal standard. The chemical shift (δ) was reported in parts per million (ppm), and the following abbreviations indicate the *J* value in Hz and the signals: s: singlet, d: doublet, t: triplet, m: multiplet, dd: double doublet, br: broad. The Inset of (**D**) shows the speculated chemical structure of garlicnin.

We successfully purified garlicnin B1 with a relatively stable and high yield using a previously-reported extraction and isolation protocol for garlic [11]. To obtain enough garlicnin B1 for in vitro and in vivo pharmacological evaluation, we repeated the extraction and purification protocol 5 times, each time using 1–2 kg of garlic. The average yield was approximately 500 mg/1 kg garlic, which is comparable to our previous report [11], indicating that the extraction and purification method for garlicnin B1 is well-established. The purified garlicnin B1 showed a single dot in TLC as compared to the standard garlicnin B1 (Figure 1B). The UV spectra of garlicnin B1 showed a maximal absorption at 248 nm (Figure 1B). Accordingly, the HPLC analysis using a UV detector at 248 nm exhibited a major peak at a retention time of 3.410 min (Figure 1C), suggesting there are no apparent degraded derivatives of garlicnin B1. To further confirm the purity of obtained garlicnin B1, we carried out ^1^H-NMR spectra; the results are as follows: ^1^H NMR δ 5.70 (1H, m, H-3′), 5.42 (1H, s, H-4′), 5.39 (1H, d, *J* = 6.9 Hz, H-3′), 5.07 (1H, br d, *J* = 3.5 Hz, H-2), 4.23 (1H, dd, *J* = 5.8, 1.8 Hz, OH), 4.10 (1H, d, *J* = 5.7 Hz, H-5), 3.63 (1H, dd, *J* = 12.6, 5.8 Hz, H-2′), 3.36 (1H, dd, *J* = 12.6, 3.4 Hz, H-2′), 2.20 (1H, m, H-4), 2.02 (1H, m, H-3), 1.29 (1H, d, *J* = 6.9 Hz, CH_3_-4), 1.08 (1H, d, *J* = 6.9 Hz, CH_3_-3). The ^1^H NMR is similar to the reported literature of standard garlicnin B1 [11]. All signals were sharp and we did not observe apparent signals of impurities (Figure 1D). According to these findings, though it is not quantitative, we considered the extraction and purification of garlicnin B1 to be successful with considerable high purity, which is suitable for the following biological analyses.

### 3.2. Suppression of Intracellular ROS Triggered by H_2_O_2_ Treatment by Garlicnin B1

**Figure 2:**
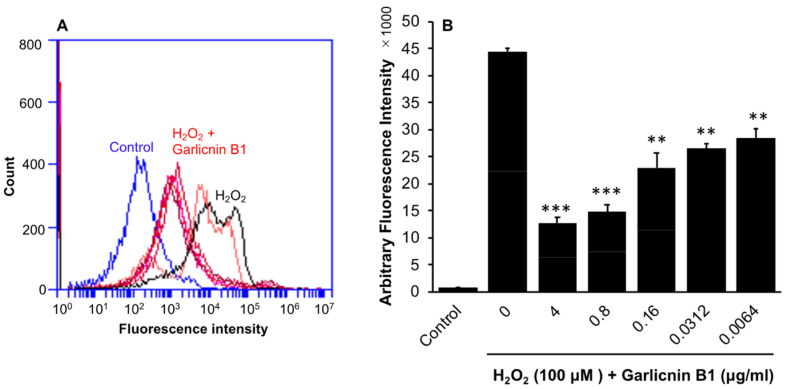
Suppression of intracellular ROS induced by hydrogen peroxide (H_2_O_2_) by treatment with garlicnin B1 in the C26 colon cancer cells. The C26 cells were treated by increasing concentrations of garlicnin B1 for 24 h before the addition of H_2_O_2_. The amount of intracellular reactive oxygen species (ROS) was then measured using the fluorescence ROS probe DCDHF-DA and analyzed by flow cytometry. (**A**) shows the histogram of flow cytometry, and (**B**) shows the quantitative results. Treatment with garlicnin B1 led to a dose-dependent reduction in intracellular ROS levels induced by H_2_O_2_. Values are means ± SD; n = 4–8. ** *p* < 0.01, *** *p* < 0.001 versus H_2_O_2_ alone group. See text for details.

To investigate the potential anti-oxidative activity of garlicnin B1, we first examined its effects in vitro, as many sulfur-containing compounds, such as glutathione (GSH), N-acetyl cysteine, and persulfides, are known for their anti-oxidant properties [17,18]. Additionally, sulfur-containing heterocyclic compounds have been reported to possess medicinal properties, including anti-oxidative and anti-inflammatory effects [24,25]. In the C26 colon cancer cells, treatment with garlicnin B1 did not significantly affect intracellular ROS levels, as indicated by fluorescence ROS probe DCDHF-DA (Supplementary Data Figure S1). However, when intracellular ROS generation was triggered by H_2_O_2_, garlicnin B1 treatment significantly inhibited intracellular ROS levels in a dose-dependent manner (Figure 2). These findings suggest that garlicnin B1 does not alter the redox homeostasis of the cells, but rather exerts potent anti-oxidative effects against oxidative stress.

### 3.3. Garlicnin B1 Exerts Remarkable Anti-Inflammatory Effect against DSS-Induced Mouse Colitis

**Figure 3:**
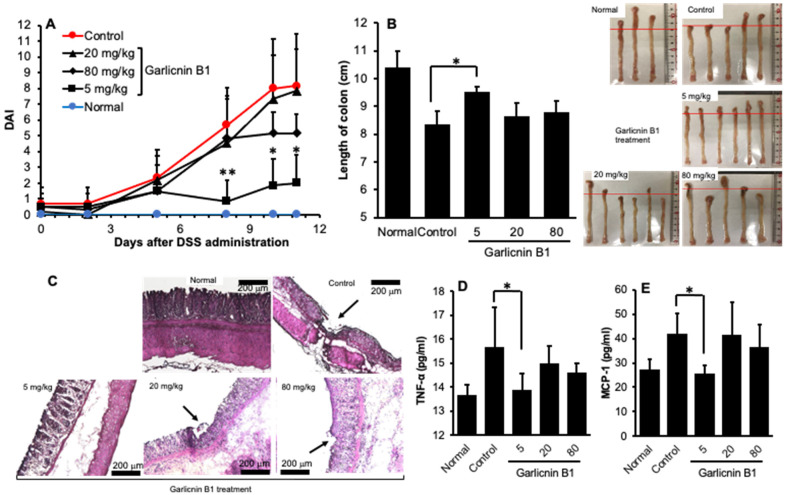
The therapeutic effect of garlicnin B1 on dextran sulfate sodium (DSS)-induced murine colitis was evaluated. The DSS-induced colitis model was established by oral administration of 2% DSS. During the experiment, colitis symptoms were recorded daily to obtain the disease activity index (DAI) values (**A**). Garlicnin B1 treatment was initiated thrice a week from the 1st day of DSS administration. On day 11, when severe colitis appeared, the mice were sacrificed, the length of the colon was measured (**B**), and a histological examination of the colon was performed (**C**). Plasma levels of inflammatory cytokines (TNF-α and MCP-1) were also measured (**D**,**E**). The arrows indicate tissue damage in the colon mucosa. Values are presented as means ± standard deviation (SD); n = 6. * *p* < 0.05, ** *p* < 0.01. Bar, 200 μm. See text for details.

Given the strong anti-oxidative effect exhibited by garlicnin B1, we anticipated its therapeutic potential in ROS-related inflammatory diseases. Therefore, we utilized a DSS-induced mouse colitis model, a commonly used model of inflammatory bowel disease known to involve excessive ROS [20,26,27]. As shown in Figure 3A, colitis symptoms such as loose stools and diarrhea were observed on day 5 after feeding 2% DSS, resulting in an elevation of DAI value. On day 11, the mice manifested severe diarrhea with bloody stools, as indicated by a significant increase in the DAI value (Figure 3A), accompanied by a remarkable decrease in body weight (Supplementary Data Figure S2). Treatment with garlicnin B1 at a dose of 5 mg/kg (three times a week) significantly improved the symptoms of colitis, as evidenced by a significant decrease in the DAI value (Figure 3A). The beneficial effect of garlicnin B1 was also supported by the pathological changes in the colon. DSS-induced colitis resulted in colon shortening (Figure 3B), and histological examination revealed tissue damage of the colon mucosa (Figure 3C, indicated by the arrow). Garlicnin B1 treatment (5 mg/kg) markedly recovered colon length, and the mucosal damage was much less than in untreated control mice, with a histological appearance similar to that of normal mice (Figure 3B,C). Additionally, we observed a significant increase in the levels of inflammatory cytokines, such as MCP-1 and TNF-α, in mice receiving DSS, while treatment with garlicnin B1 (5 mg/kg) significantly suppressed these inflammatory cytokine levels (Figure 3D,E). These findings are consistent with the improved symptoms and pathological changes of colitis, further supporting the anti-inflammatory activity of garlicnin B1.

However, we unexpectedly discovered that the anti-inflammatory effect of garlicnin B1 is not dose-dependent. In other words, at higher doses (i.e., 20 and 80 mg/kg), we did not observe any further beneficial outcomes. On the contrary, we found that colitis worsened compared to treatment with 5 mg/kg of garlicnin B1, as evidenced by an increased DAI value (Figure 3A) and exacerbation of colon pathology (Figure 3B,C), as well as an elevation in inflammatory cytokines (Figure 3D,E). These findings suggest that garlicnin B1 may have different roles depending on its concentration, where it exhibits anti-oxidative and anti-inflammatory roles at low and moderate concentrations, but may induce tissue damage and cell death at high concentrations. Therefore, we further investigated the cytotoxic or tumoricidal effects of garlicnin B1 using cultured C26 colon cancer cells.

### 3.4. In Vitro Cytotoxicity of Garlicnin B1

**Figure 4:**
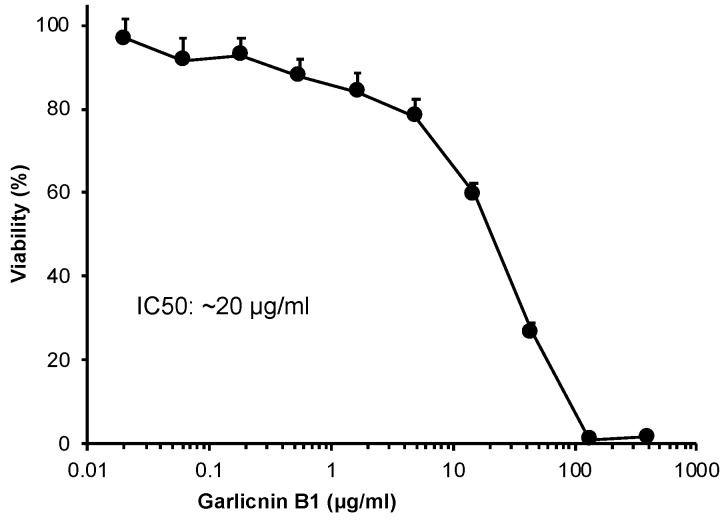
In vitro cytotoxicity of garlicnin B1 in the C26 colon cancer cells. The C26 cells were exposed to increasing concentrations of garlicnin B1 for 24 h, and cell viability was then determined by the MTT assay. Data are presented as means ± standard deviation (SD); n = 8. See text for details.

The results in Figure 4 demonstrate that there was no significant cytotoxicity of garlicnin B1 against the C26 colon cancer cells at concentrations up to 5 μg/mL. However, when the concentration exceeded 10 μg/mL, strong cytotoxicity was observed with an IC50 of approximately 20 μg/mL. At a concentration of 100 μg/mL, garlicnin B1 was able to eliminate all cancer cells. These findings suggest that garlicnin B1 has the potential to exert a strong anti-cancer effect if used appropriately.

### 3.5. Garlicnin B1 Exhibits Potent Anti-Cancer Effect in AOM/DSS-Induced Colon Cancer Model

**Figure 5:**
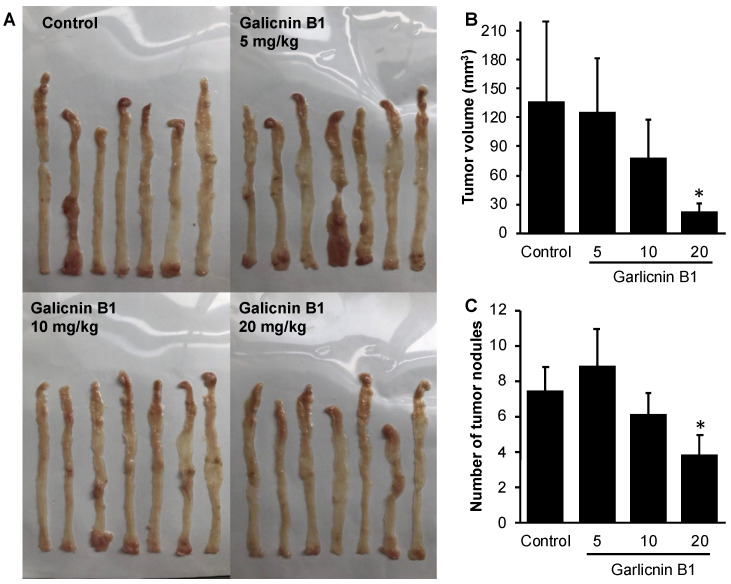
Inhibitory effect of garlicnin B1 on colon carcinogenesis in mice treated by azoxymethane (AOM)/dextran sulfate sodium (DSS). (**A**) shows representative pictures of the colon from different experimental groups, (**B**) shows the cumulative size of tumor nodules in each mouse, and (**C**) shows the average number of tumor nodules in the colon. Values are presented as means ± standard deviation (SD); n = 7. * *p* < 0.05 versus the untreated control group. See text for details.

To investigate the anti-cancer effect of garlicnin B1, we utilized an AOM/DSS-induced colon cancer model, which is a carcinogen-induced cancer model that more closely resembles naturally formed colon cancer. Garlicnin B1 treatment was initiated at the onset of colon cancer by AOM and continued throughout the experiment period. The results, as shown in Figure 5, indicate that garlicnin B1 treatment significantly decreased the number and size of tumor nodules in a dose-dependent manner. In contrast to the anti-inflammatory effect of garlicnin B1 observed in the DSS-induced colitis model (Figure 3), 5 mg/kg of garlicnin B1 did not exhibit apparent tumor suppression (Figure 5). These findings suggest that the anti-cancer effect of garlicnin B1 is mainly due to its tumoricidal effects (Figure 4) rather than a cancer-preventive effect through its anti-inflammatory activity.

### 3.6. Garlicnin B1 Exhibits Potent Cancer Therapeutic Effect in S180 Solid Tumor Model

To further confirm the therapeutic effect of garlicnin B1 against cancer, we utilized the S180 transplanted tumor model, a commonly used solid tumor model in which tumor cells are inoculated into the dorsal skin of mice to form cancer. In this model, we administered garlicnin B1 treatment when the tumor had formed and reached a palpable size (7–8 mm in diameter) to evaluate the therapeutic effect of garlicnin B1.

**Figure 6:**
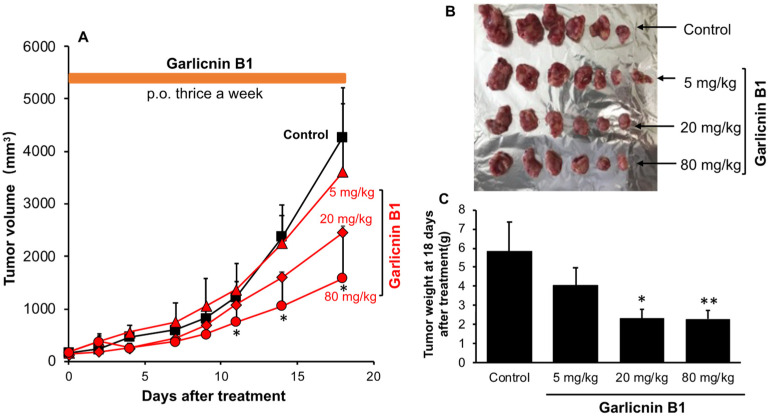
Anti-tumor effect of garlicnin B1 against mouse sarcoma S180 solid tumor. Garlicnin B1 treatment was initiated on day 7 after S180 tumor cell inoculation when tumors had grown to 7–8 mm in diameter. (**A**) shows the changes in tumor volume during the experiment, (**B**) shows representative pictures of tumors collected on day 18 after garlicnin B1 treatment, and (**C**) shows the average tumor weight of collected tumors. Data are presented as means ± standard deviation (SD); n = 4–8. * *p* < 0.05; ** *p* < 0.01 versus the untreated control group. See text for details.

As expected, similar to the results in the AOM/DSS colon cancer model (Figure 5), we observed a dose-dependent cancer-suppressing effect, with significant anticancer activity achieved at 20 mg/kg and 80 mg/kg doses. At these doses, the tumor size/weight was reduced by more than 50% compared to the untreated control group (Figure 6). These findings support our notion that garlicnin B1 primarily exhibits cancer therapeutic activity.

## 4. Discussion

In this study, we successfully developed an extraction and purification method for garlicnin B1 with high yield, throughput, and considerable purity of the final product (Figure 1). In vitro studies demonstrated the potent anti-oxidative activity of garlicnin B1 (Figure 2), which partly contributes to its anti-inflammatory effect, as shown by the DSS-induced mouse colitis model (Figure 3). Additionally, garlicnin B1 exhibited a significant tumoricidal capacity (Figure 4), leading to a marked anti-cancer effect observed in both the tumor xenograft model and the carcinogen-induced colon cancer model (Figure 5 and Figure 6). These findings suggest that garlicnin B1 has multifarious functions and applications in different scenarios.

One important and intriguing finding of this study is the dose–response effect of garlicnin B1. In the DSS-induced colitis model, a relatively low concentration of garlicnin B1 (5 mg/kg) demonstrated a significant anti-inflammatory tissue protective effect, greatly improving the symptoms and pathological changes of colitis. However, a further increase in the dose did not show any beneficial effect but rather exacerbated the progression of colitis (Figure 3). Conversely, at higher doses (20 and 80 mg/kg), garlicnin B1 exhibited strong cytotoxicity and anti-cancer effects (Figure 5 and Figure 6). These results suggest a U-shaped dose-effect of garlicnin B1, whereby low or moderate doses mainly exhibit anti-inflammatory effects, while high doses may trigger pro-inflammatory reactions and cytotoxic cascades leading to cell death and tissue damage. This U-shaped or convex U-shaped dose effect is a common biological event observed in chemicals with hormonal activities and natural products [28]. We have previously observed similar dose effects in our studies of Kumaizasa bamboo leaf extract, as well as extracts of *Phellinus linteus*, Bamboo (*Sasa senanensis*) Leaf, and Chaga Mushroom (*Inonotus obliquus*) [22,29].

Considering the unique dose–response effect of garlicnin B1, it is critical to carefully consider and manipulate it when translating and applying it clinically. While garlicnin B1 demonstrates diverse pharmacological functions that could be utilized to treat different diseases and disorders, such as inflammation and cancer, its U-shaped dose–response must be taken into account. When developing garlicnin B1 as a drug candidate, its dose–response activity must be evaluated and used in different dose settings depending on the disease and pathological scenarios. For example, when treating inflammatory diseases, overdosage should be avoided to prevent triggering reverse effects. Conversely, when developing garlicnin B1 as a candidate drug for cancer, higher dose settings are necessary to achieve therapeutic effects. Inadequate doses may result in insignificant therapeutic effects and may even promote tumor growth by suppressing anti-cancer innate immunity and inflammatory responses.

Regarding the clinical translation of garlicnin B1, several obstacles must be addressed, with stability being the most critical issue. While garlicnin B1 can be dissolved in aqueous solutions, it rapidly decomposes and becomes inactive in this condition. It is relatively stable in some organic solvents, such as acetone, DMSO, and chloroform, but these solvents are not suitable for human use or in vivo applications. In this study, we used methyl cellulose as the solvent for garlicnin B1, similar to our previous study of onionin A1 [12,13,14]. Although methyl cellulose is a stable and safe solvent commonly used as a food additive, its high viscosity limits its practical application, especially for systemic administration. Therefore, it is essential to develop a stable drug delivery system for garlicnin B1. Additionally, as garlicnin B1 is a small molecule, it is rapidly cleared from the body and has no disease-targeting effects, such as tumor targeting. Nano-formulation of garlicnin B1 is a promising strategy to overcome these limitations. We previously developed a polymeric nano-micelle system using styrene maleic copolymer to deliver highly unstable carbon monoxide-releasing molecules (SMA/CORM2) [20]. Due to micelle formation, SMA/CORM2 exhibited high water solubility and stability in aqueous solutions, resulting in prolonged plasma half-life and high bioavailability [20]. Moreover, the large size of the nano-micelle enabled it to selectively accumulate in highly permeable blood vessels in inflammatory tissues, exploiting the enhanced permeability and retention (EPR) effect [30,31,32]. Further studies will focus on developing a nano-designed drug delivery system for garlicnin B1.

This study provides proof of concept for the anti-inflammatory and anti-cancer effects of garlicnin B1, the most abundant active cyclic sulfide in garlic. The results from different mouse models strongly support our expectations. While we did not investigate the mechanisms of action in depth in this study, follow-up studies will focus on this issue. Based on the preliminary results of the anti-oxidative activity of garlicnin B1 (Figure 2) and previous related literature, we propose the following directions for future studies.

Regarding the anti-oxidative effect of garlicnin B1, although we revealed here the potent ROS inhibiting activity of garlicnin B1, it is still not clear and needs to be clarified whether garlicnin B1 exhibits the anti-oxidative effect directly or upon upregulation anti-oxidative molecules, e.g., GSH, catalase, superoxide dismutase, and heme oxygenase-1. In the case of the latter, how does garlicnin B1 increase the expression/activity of those molecules? The answers to these questions will much helpful to understand the anti-oxidative nature of garlicnin B1.

As for the anti-inflammatory effect of garlicnin B1, besides its anti-oxidative activity, we consider that macrophage reprogramming and inflammasome are two interesting aspects worthy of further investigation. It is commonly known that the activation/polarization of macrophages plays important roles in the pro- and anti-inflammatory responses of the host to various stimuli. Activation of pro-inflammatory M1 macrophages promotes the progression of inflammation, while polarization toward M2 macrophages triggers an opposite anti-inflammatory protective response, which has been verified by numerous studies including our own [33,34,35]. A recent study also suggested that in a non-alcoholic steatohepatitis model, diallyl disulfide suppressed the progression of inflammation, partly through the inhibition of macrophage activation [36]. Thus, it is reasonable to expect that garlicnin B1, as an active cyclic disulfide, may trigger macrophage polarization toward the anti-inflammatory M2 phenotype. Additionally, the NLRP3 (NOD-, LRR-, and pyrin domain-containing protein 3) inflammasome is a well-studied protein complex involved in the progression of numerous inflammatory diseases, including DSS-induced colitis [37,38,39]. Recently, Sawa’s group provided evidence suggesting the inhibitory effect of reactive persulfides on NLRP3 inflammasome activation [18], proposing an alternative mechanism of the anti-inflammatory action of garlicnin B1.

Regarding the anti-cancer properties of garlicnin B1, suppression of the expression of TAM and immunosuppressive cells, such as myeloid-derived suppressor cells (MDSCs), has been reported as an essential mechanism of active garlic sulfides, such as onion A1 [12,13]. Moreover, in this study, we observed relatively potent cytotoxicity of garlicnin B1 (~20 μg/mL). Previous studies using Allium extracts showed that many active sulfur compounds similar to garlicnin B1 exhibited considerable cytotoxicities against various tumor cancer cells, including leukemia cells and colon cancer cells, having IC50 values between 10 μg/m and 100 μg/m [40,41,42]. Our results are consistent with previous literatures, suggesting direct tumoricidal activity of garlicnin B1. Moreover, Osipova et al. reported that aromatic oligosulfides induce apoptosis of cancer cells [43]. In future studies, we will investigate the possible mechanisms of the tumoricidal activity of garlicnin B1, focusing on apoptosis as well as other forms of cell death, such as ferroptosis and autophagy.

Taken together, we have demonstrated here the anti-inflammatory and anti-cancer effects of garlicnin B1, the most abundant garlic sulfide, in various inflammatory and solid tumor models. By carefully manipulating the dosing regimen, different effects can be achieved. The multifunctional properties of garlicnin B1 make it highly useful and applicable. With further elucidation of its mechanisms of action, we anticipate that garlicnin B1 could be developed as a candidate drug for the treatment of cancer and inflammatory diseases.

## Data Availability

Data is contained within the article.

## Supplementary Materials

The following supporting information can be downloaded at: https://www.mdpi.com/article/10.3390/antiox12040869/s1, Figure S1: Effect of garlicnin B1 on intracellular ROS in the C26 colon cancer cells; Figure S2: Body weight changes of the mice during DSS-induced colitis in the absence/presence of garlicnin B1 treatment.
