# Supplementary material for: Garlicnin B1, an Active Cyclic Sulfide from Garlic, Exhibits Potent Anti-Inflammatory and Anti-Tumor Activities
Source: Antioxidants (Basel). 2023 Apr 3;12(4):869. doi: 10.3390/antiox12040869 (PMC10135383; doi:10.3390/antiox12040869)
Supplement: Supplementary file 1 [file antioxidants-12-00869-s001.zip › antioxidants-2302617-supplementary.pdf]

antioxidants

MDPI

Article

Garlicnin B1, an Active Cyclic Sulfide from Garlic, Exhibits Potent Anti-Inflammatory and Anti-Tumor Activities

Shanghui Gao [1], Kai Yang [1,2], Toshihiro Nohara [1], Tsuyoshi Ikeda [1], Jian-Rong Zhou [1], Kazumi Yokomizo [1,*] and Jun Fang [1,*]

[1] Faculty of Pharmaceutical Sciences, Sojo University, Kumamoto 860-0082, Japan; gaoshanghui94@gmail.com (S.G.); yangkai@ahyz.edu.cn (K.Y.); none@ph.sojo-u.ac.jp (T.N.); tikeda@ph.sojo-u.ac.jp (T.I.); zhoujr@ph.sojo-u.ac.jp (J.-R.Z.)
[2] Department of Medical Technology, Anhui Medical College, No.632, Furong Road, Hefei 230601, China
* Correspondence: yoko0514@ph.sojo-u.ac.jp (K.Y.); fangjun@ph.sojo-u.ac.jp (J.F.); Tel.: +81-(96)-3264091 (K.Y.); +81-(96)-3264137 (J.F.)

Supplementary materials

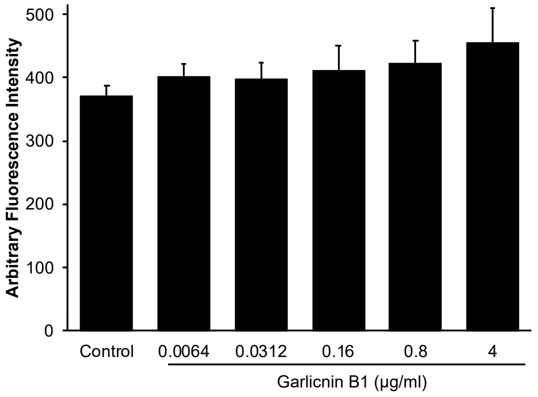

Figure S1. Effect of garlicnin B1 on the production of intracellular ROS in C26 colon cancer cells. C26 cells were treated by increasing concentrations of garlicnin B1 for 24 h. The amount of intracellular reactive oxygen species (ROS) was then measured using the fluorescence ROS probe DCDHF-DA and analyzed by flow cytometry. See text for details.

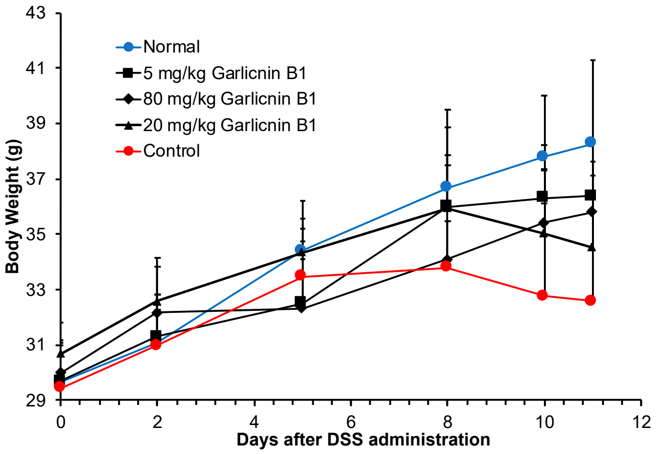

Figure S2. Body weight changes of the mice exposed to dextran sulfate sodium (DSS) with/without garlicnin B1 treatment. See text for details.
